# A novel simple traumatic brain injury mouse model

**DOI:** 10.1186/s41016-022-00273-5

**Published:** 2022-04-01

**Authors:** Chen Chen, Jiawei Hou, Junfeng Lu, Zeyu Zhu, Yang Yang, Weijia Peng, Rongbiao Pi

**Affiliations:** 1 School of Pharmaceutical Sciences, Sun Yat-Sen University, Guangzhou, 510006 China; 2 School of Medicine, Sun Yat-Sen University, 132# Waihuandong Road, High Education Mega Center, Guangzhou, 510006 P. R. China; 3 International Joint Laboratory (SYSU-PolyU HK) of Novel Anti-Dementia Drugs of Guangzhou, Guangzhou, China

**Keywords:** Traumatic brain injury, Blood-brain barrier, Rubber bullet impact model, Cognitive dysfunction, Tau phosphorylation

## Abstract

**Background:**

Traumatic brain injury, one of the leading causes of death in adults under 40 years of age in the world, is frequently caused by mechanical shock, resulting in diffuse neuronal damage and long-term cognitive dysfunction. Many existing TBI animal models revival with expensive equipment or special room are needed or the processes of operations are complex and not easy to be widely used. Therefore, a simpler TBI model needs to be designed.

**Methods:**

Our TBI model is an innovation of the modeling method through air guns shutting rubber bullets. A core facet is the application of our designed rubber bullet impact device. It could focus the hitting power to the fixed site of the brain, thus triggering a mild closed head injury. Moreover, the degree of damage can be adjusted by the times of shots.

**Results:**

Our model induced blood-brain barrier leakage and diffused neuronal damage. Besides, it led to an increased level of Tau phosphorylation and resulted in cognitive dysfunction within several weeks post-injury.

**Conclusion:**

Our TBI model is not only simple and time-saving but also can simulate mild brain injuries in clinical. It is suitable for exploring pathobiological mechanisms as well as a screening of potential therapies for TBI.

**Supplementary Information:**

The online version contains supplementary material available at 10.1186/s41016-022-00273-5.

## Background

Traumatic brain injury (TBI) refers to the organic injury of brain tissue caused by violence on the head, accompanied by a series of clinical symptoms, including headache, vomiting, disturbance of consciousness, changes of vital signs, etc., TBI mainly divided into concussion, contusion, and laceration of brain, diffuse axonal injury, and brain stem injury [1]. The most common injury mechanisms in the order of frequency from high to low are accidental falls, accidental object strikes, traffic accident injuries, attacks, and some unspecified mechanisms. Intentional self-injury and gunfire injuries are the most important causes during wartime [2]. TBI is one of the leading causes of death among young people, accounting for about half of all trauma-related deaths in the world. The World Health Organization estimates that 150 to 300 out of every 100,000 people worldwide are affected by TBI [3]. TBI also has significant social and financial costs, with the annual financial burden associated with TBI estimated at $9–10 billion, which has brought a heavy burden to the society and families and has become a worldwide public health problem [4]. To further study the pathophysiological mechanism and treatment of TBI, numerous animal models of TBI have been developed since the 1980s.

At present, the following models are widely used in experimental research: weight-drop injury models [5–7], fluid percussion injury models [8–11], controlled cortical impact injury models [12–15], blast-induced traumatic brain injury models [16–18], and penetrating ballistic-like brain injury models [19], etc. Although the existing models of TBI mimic most of the histopathological and functional results observed in clinical, they cannot fully replicate human TBI. At the same time, these modeling methods often require pre-injury manipulations on the skull bone or special complex devices, which not only require long-term maintenance but are expensive. To meet the demand for a simpler and cost-effective device, herein we introduce a new TBI model via a rubber bullet impact without pre-injury manipulations on the skull bone.

## Methods

### Animals

Male C57BL/6 mice (8 weeks of age) were purchased from Sun Yat-Sen University Laboratory Animal Center. All mice were housed in a temperature (20 ± 2 °C) and humidity-controlled room with a 12-h dark/light cycle with food and water available ad libitum. All procedures adhere to the National Institutes of Health guidelines and were approved by the Ethics Committee on the Care and Use of Laboratory Animals of Sun Yat-Sen University (Guangzhou, China) (No. East-C2020-0250XS).

### Device instructions

**Fig. 1:**
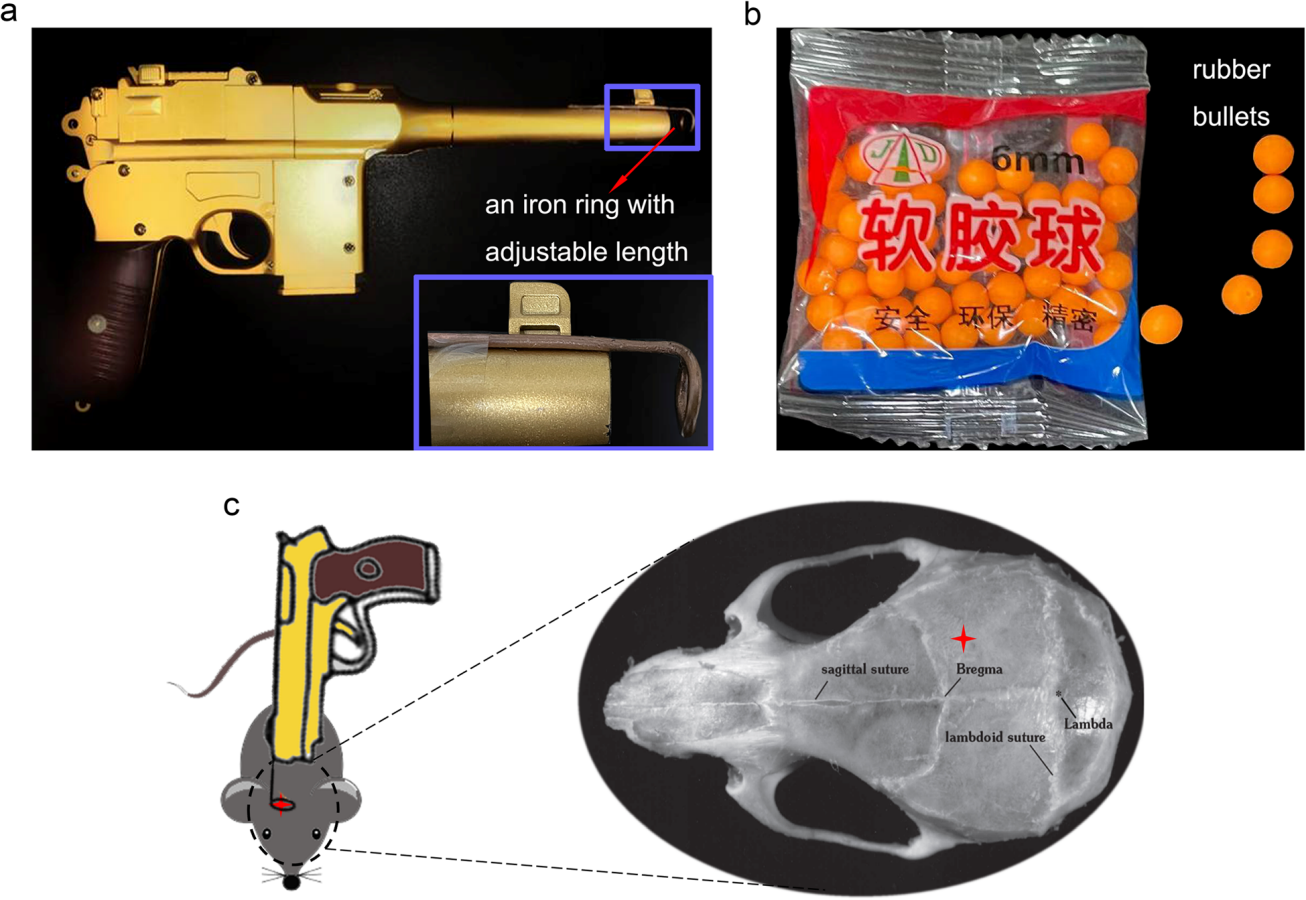
The TBI device and hit point*.* **a** The device consists of an air gun and an iron ring with adjustable length attached to the muzzle. **b** Rubber bullets (size: 6 mm in diameter). **c** The hit point on the right hemisphere (3 mm lateral and 1 mm posterior to the bregma)

The new TBI device and its accessory components are shown in Fig. 1. It consists of an air gun (F308A, Yiwu Lasalle Toys Co., Ltd.) fixed on an iron ring with adjustable length attached to the muzzle and the rubber bullets (Fig. 1a, b). After exposing the skull, locate the impact area, and fire a rubber bullet from the trigger of an air gun to establish the model (Fig. 1c). Researchers can adjust the distance between the muzzle and the skull to produce different degrees of damage by adjusting the length of the iron ring.

### Animal experimental protocol

The timeline of the experimental design was described as Additional file 1.

#### Preparation of TBI device

1. Assemble the TBI device, install and fix an iron ring with adjustable length on the muzzle of the air gun.
  ▲ Critical step: the iron ring with adjustable length is necessary for the TBI device. Researchers can adjust the distance between the muzzle and the skull to produce different degrees of damage by adjusting the length of the iron ring.
2. Check the TBI device to ensure that the barrel of the air gun is smooth and that rubber bullets can be fired properly.

▲ Critical step: calibrating the device before the start of the operation is very important to ensure its smooth and accurate operation.

#### Surgical preparation

**Fig. 2:**
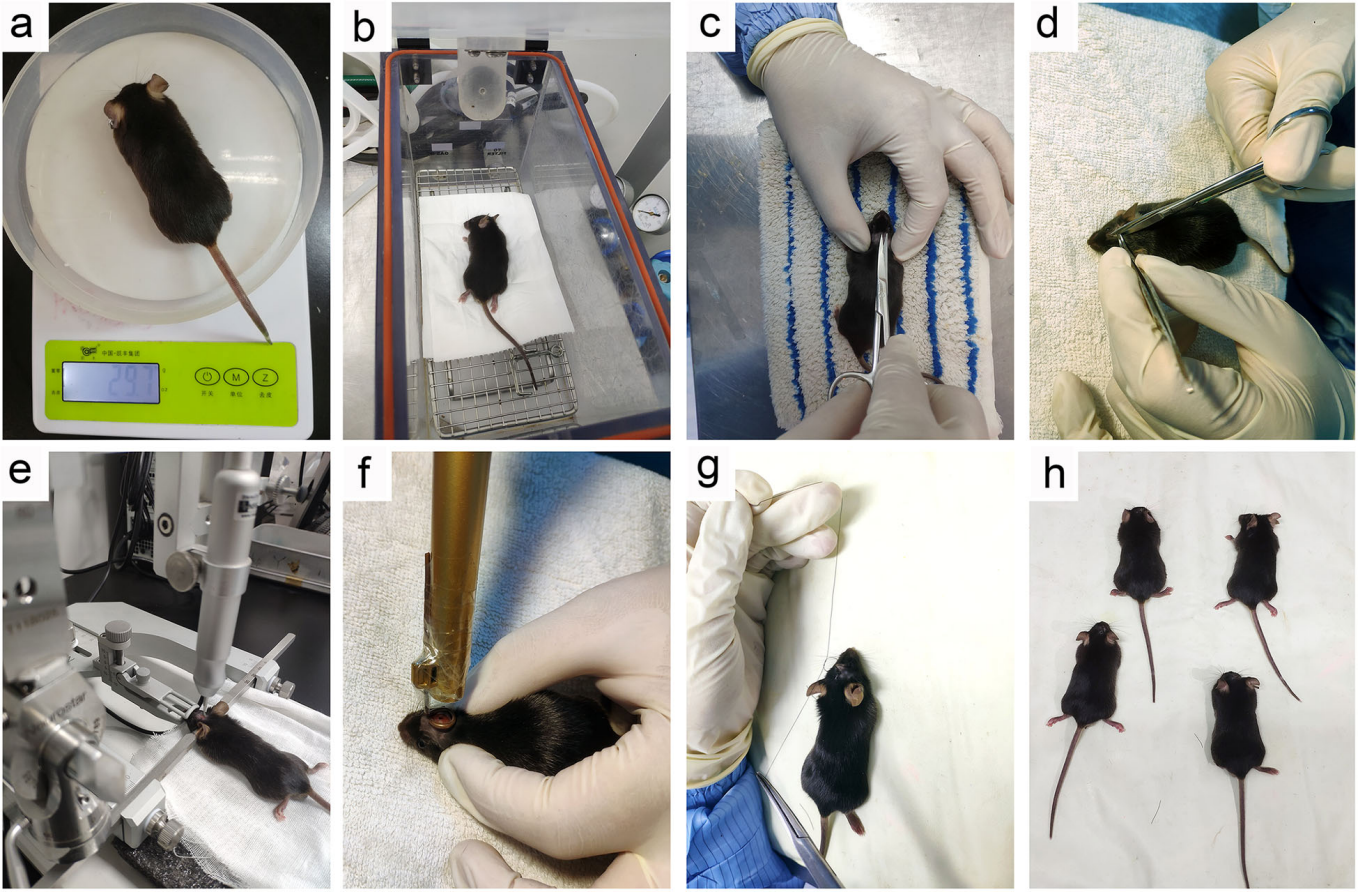
TBI modeling flowchart. **a** Weight the mice. **b** Anesthesia. **c** Cut the hair off the surgical area. **d** Expose the skull. **e** Position the impact area on the right hemisphere. **f** Aim the iron ring of the muzzle at the impact area and fire. **g** Clean the wound and suture it quickly. **h** Recover on the electric heating pad for 1 h

3| Weight the mice to determine the amount of anesthetic needed and anesthetized with 1% pentobarbital sodium (50 mg/kg, i.p.) (Fig. 2a, b).
  **!** Caution: before handing animals, wear surgical clothes and gloves, and keep the surgical area sterile. Sterilize all surgical instruments with a high-pressure sterilizer before the operation.
4| Cut the hair off the surgical area on the head of the mice (Fig. 2c).

#### **Exposure**

5| Swab back and forth with iodine to disinfect the scalp.
6| Make a 15-mm longitudinal midline incision with an anatomical knife to expose the skull (Fig. 2d).
  ▲ Critical step: Proper exposure of the skull is necessary for locating the impact point of the rubber bullet.
  ! Caution: on the premise of meeting the positioning needs, the incision of the scalp should be completed at one time and as small as possible, which is beneficial to the wound recovery of animals.
7| The mice were fixed on the brain stereotaxic instrument (Neurostar, NE13008) and position the impact point (3 mm lateral and 1 mm posterior to the bregma) (Fig. 2e).

#### Induction of trauma

8| Put the mice on the foam cushion.
  ▲ Critical step: the foam cushion is necessary to avoid damage to other parts of the mice.
9| Aim the iron ring (length: 1 cm) at the impact point, and fire one shot vertically down. The diameter of the rubber bullet (Yiwu Lasalle Toys Co., Ltd.) was 6 mm and its mass was 125 ± 5 mg, the speed at which the bullet is fired is about 30 m/s (Fig. 2f).
  ▲ Critical step: researchers need to determine the length of the iron ring and the number of impacts according to the severity of nerve trauma that best suits their scientific goal.
  ! Caution: the shooting angle should be strictly controlled.

#### Closure and postoperative care

10| Clean the wound and suture it quickly (Fig. 2g).
11| After the trauma, let the mice recover on the constant temperature heating pad (DEWEIBIO, JRD001) for 1 h (Fig. 2h).
  ▲ Critical step: maintaining temperature is necessary to reduce mice mortality after trauma.
12| Transfer the mice to the cages and raise them in a room with controllable temperature (20 ± 2 °C) and humidity. The dark/light cycle was 12 h, and food and water could be provided at will.

### Permeability of the blood-brain barrier (BBB)

Colorimetry detection of extravasated Evans Blue (EB) dye was used to evaluate the BBB permeability of the mice after TBI quantitatively, as we previously reported [20]. Briefly, 1 h before sacrifice, EB dye (BIOSHAP, BS177, freshly dissolved in sterile saline, 2% w/v in PBS) was injected into the tail vein (4 mL/kg). After about 1 h, mice were deeply anesthetized with 1% pentobarbital sodium (50 mg/kg, i.p.). Then, it was rapidly perfused from the left ventricle to the right atrium using normal saline until the effluent was clarified. Brains were quickly removed. Afterward, the blood was washed on the surface with 0.9% NaCl. The tissues in the injured hemisphere were put in formamide (10 mL/g) for homogenization, and the brains were incubated for 24 h at 60 °C water bath. Then, the homogenate was centrifuged at 4000 rpm for 20 min at 4 °C. Fluorescence values of the supernatant were analyzed by a fluorescence spectrophotometer at the wavelength of 632 nm. The contents of EB dye were quantified from a linear standard curve.

### Morris water maze

The Morris water maze test assesses spatial learning and memory function. As was previously reported, mice were subjected to four trials per day for five consecutive days in a circular pool (120 cm diameter and 50 cm high) containing a 10-cm-diameter hidden platform, which was placed in the target quadrant and submerged 1 cm below the water surface for all trials. Each mouse was placed into the water, facing the pool wall, and given 60 s to locate the hidden platform. If the mice fail to find the platform within 60 s, it would be gently guided to the platform and allowed to remain for 15 s. The time and distance to reach the platform (escape latency) were measured, which could reflect the function of spatial learning. The number of times the mice crossed the platform area and the percentage of time spent in the target quadrant would be recorded by a digital video camera (Shanghai Jiliang Software Technology Co., Ltd., DigBhv-MG).

### HE staining

The slices in the injured hemisphere were rehydrated in decreasing concentrations of ethanol (100%, 95%, 70%, and 50% (v/v)). Stain the slices with hematoxylin and eosin staining kit (Beyotime, C0105S), counterstain them in 2.5% (w/v) eosin, dehydrate them, and cover them with coverslips. Pictures of the slices were taken by a microscope (Leica, DM6B).

### Western blotting

The tissues in the injured hemisphere were homogenized with lysis buffer containing protease and phosphatase inhibitor. Then, the homogenates were centrifuged at 12,000 rpm for 15 min at 4 °C and the concentrations of protein were quantified with BCA assay. Protein samples were separated by using 10% SDS-PAGE and then transferred to a polyvinylidene fluoride membrane and incubated with 5% BSA at room temperature for 2 h. Then the membranes were incubated with the primary antibodies (rabbit monoclonal antibody to Tau (phospho S396) (Abcam, ab109390), rabbit monoclonal antibody to Tau (phospho T231) (Abcam, ab151559), and mouse monoclonal antibody to GAPDH (Sigma-Aldrich, G8795)) overnight at 4 °C. Then the membranes were washed with a tris-buffered saline/t buffer for 30 min and then incubated with secondary antibodies at room temperature for 1.5 h. The protein bands were visualized by using an ECL Prime kit (Millipore, P90719). Three independent experiments were implemented.

### Statistical analysis

The data were expressed as means ± S.E.M. for at least 3 experiments. Statistical differences between the groups were analyzed using Student’s *t* test or one-way analysis of variance (ANOVA), followed by Tukey’s post hoc test. The significance of the results was determined at *P* < 0.05.

## Results

### The mortality rate of TBI models

The basis of mortality rates assessed up to 24 h after the trauma is a useful tool for measuring the injury severity of TBI [21]. In this research, we choose the injury condition as the distance from the muzzle to the skull which was 1 cm, and the size of the rubber bullet was 6 mm once impacted. Thirty-seven mice were used to establish the TBI model, 5 mice died, and the mortality rate was 7.4% which assessed up to 24 h after trauma.

### The BBB was broken in the injured hemisphere

**Fig. 3:**
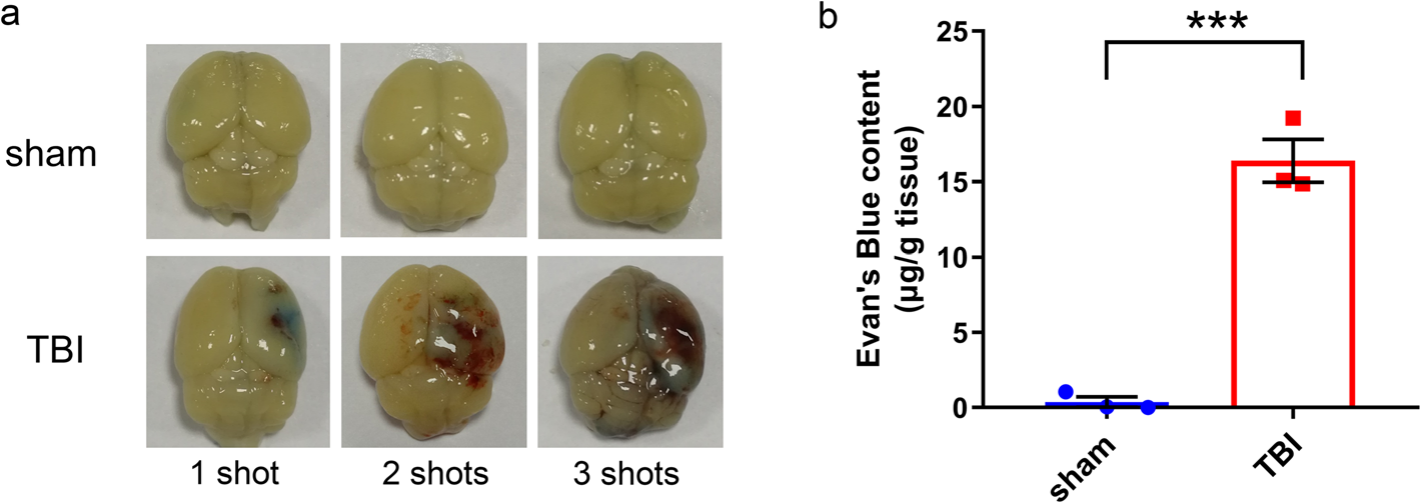
The effect of rubber bullet impact induced TBI on the permeability of BBB. **a** Representative brain pictures of the mice in TBI and sham group. The 3 brains of TBI mice were who survived 1 shot (mild, left), 2 shots (moderate, middle), and 3 shots (severe, right) respectively. **b** The content of Evans Blue in the impacted cortex of the mTBI with 1 shot and sham group. Data are presented as the mean ± S.E.M. and analyzed using the Student’s *t* test. *n* = 3/group. ***P < 0.001 vs. sham group

The effect of rubber bullet impact induced TBI on the permeability of BBB was evaluated by EB extravasation using spectrophotometry 48 h after the injury. Naked eye observation showed that brain edema and hemorrhage appeared in the hit area of mice in the TBI injury group 48 h after injury (Fig. 3a). The content of Evans Blue in the brain on the same side as the hit area is significantly higher in the TBI group than in the sham group (*P* < 0.001, Fig. 3b). It indicated that the permeability of BBB was destroyed 48 h after TBI.

### Cognitive disorder in the Morris water maze

**Fig. 4:**
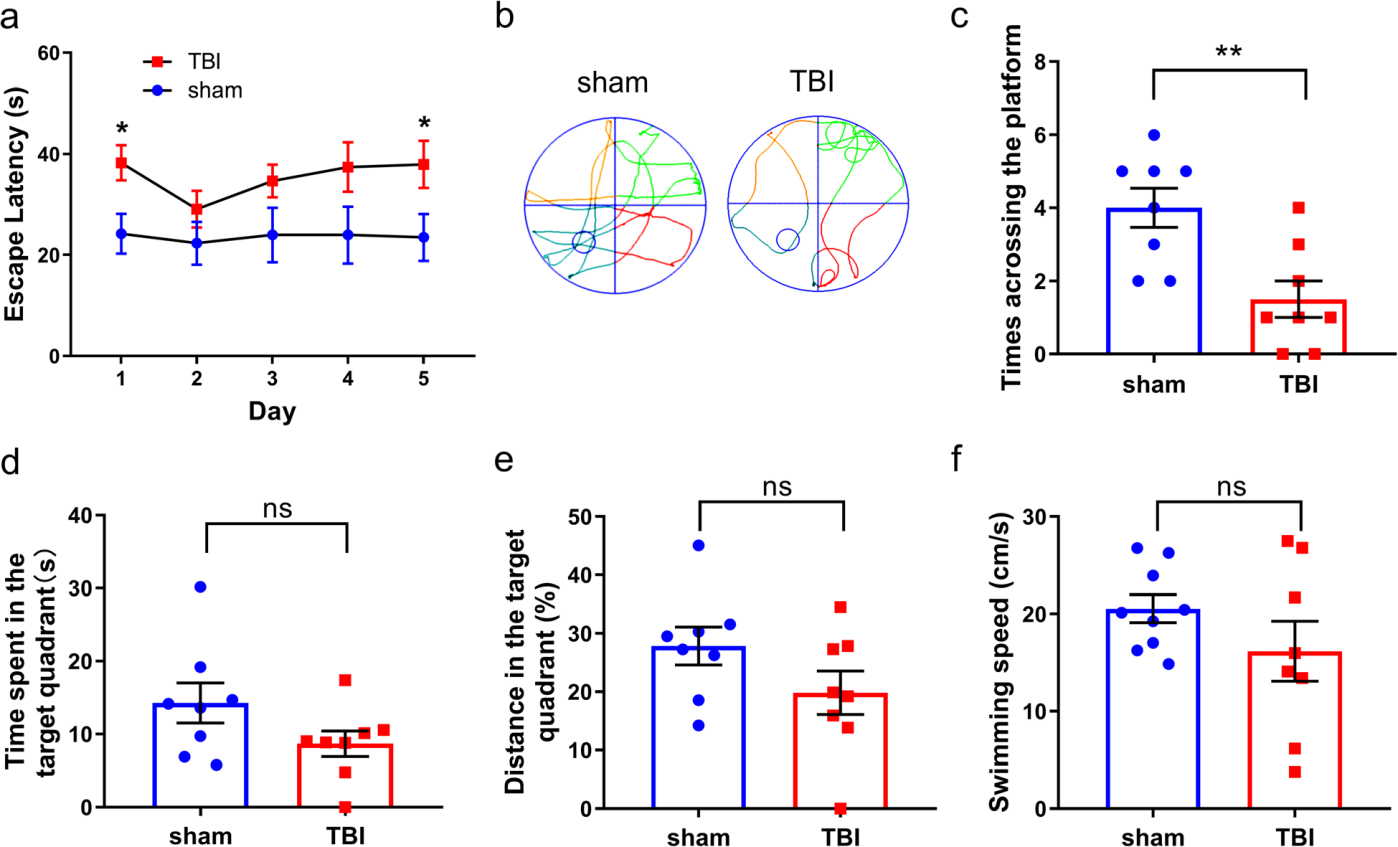
The effects of rubber bullet impact induced mTBI on the learning and memory of mice. **a** Mean escape latency of the mice in the positioning trials, which were conducted for 5 consecutive days. **b** Representative swimming tracks of the mice in the space exploration experiment. The circle in the third quadrant represented the location of the hidden platform. **c** The times crossing platform of the mice in the space exploration experiment. **d** The time spent in the target quadrant of the mice in the space exploration experiment. **e** The distance (%) in the target quadrant of the mice in the space exploration experiment. **f** Swimming speed of mice in the space exploration experiment. Data are presented as the mean ± S.E.M. and analyzed using Student’s *t* test and multiple *t* tests. *n*=8–9/group. **P < 0.01, *P < 0.05 vs. sham group

The cognitive ability of mice was tested by the Morris water maze test. Evaluation of cognitive deficits in injured mice 14 days after TBI using the Morris water maze revealed that TBI impairs the spatial memory and learning ability of mice (Fig. 4). In the continuous 5-day positioning trials, the escape latency of the TBI group was significantly longer than that of the sham group on the 1st and 5th day (*P* < 0.05, Fig. 4a). In the space exploration experiment, there was no significant difference in the average speed between the two groups (Fig. 4f), indicating that the exercise ability of mice recovered 20 days after TBI. Compared with the sham group, the number of times of crossing the platform in the TBI group decreased significantly (*P* < 0.01, Fig. 4c). The percentage of distance and the time spent in the target quadrant in the TBI group tended to decrease, but there was no significant difference (Fig. 4d, e).

### Neuronal injures in the cortex of injured hemisphere

**Fig. 5:**
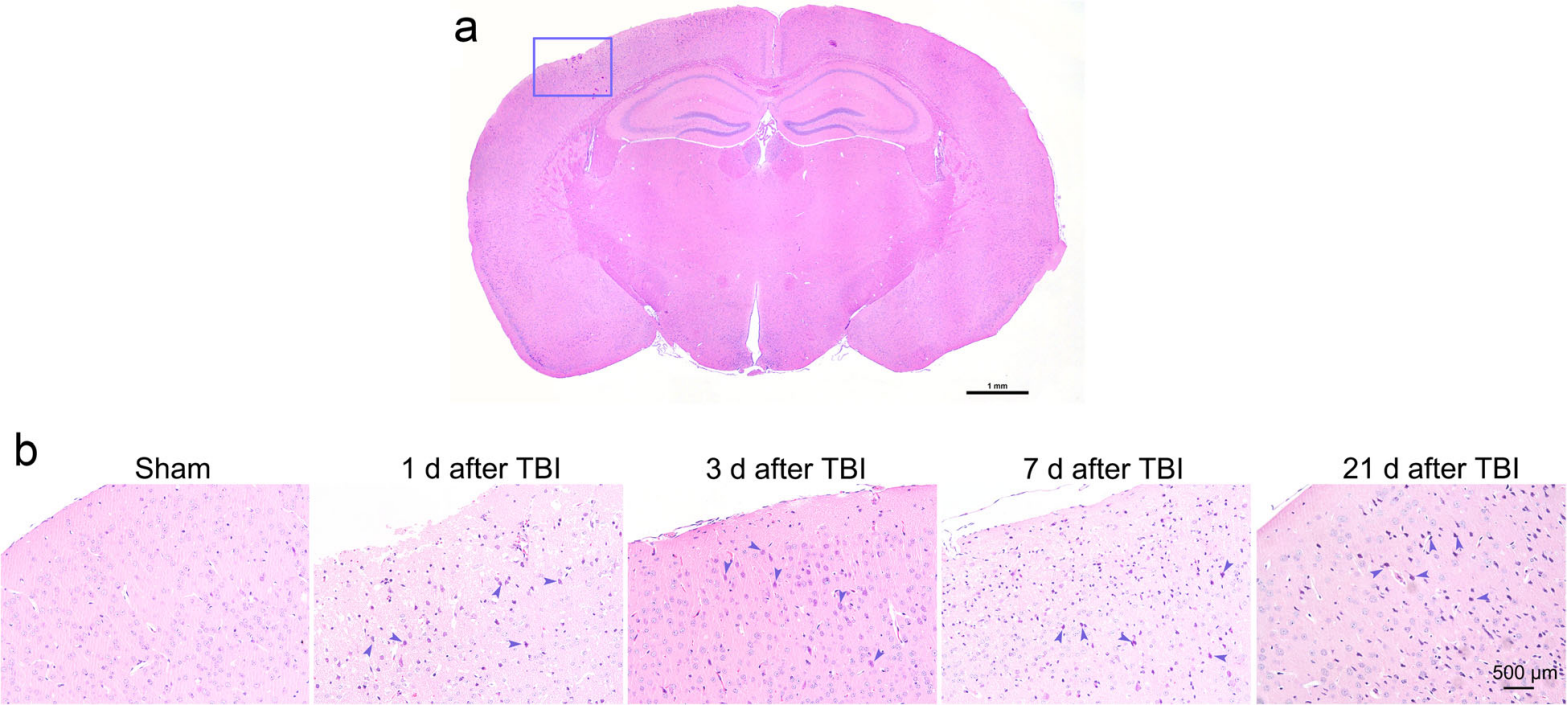
The effect of rubber bullet impact induced mTBI on the morphology of cortical neurons. **a** A representative area of the impacted hemisphere. The scale bar represents 1 mm. **b** The nuclei of cortical neurons in the injured hemisphere were dark orchid which was marked by the blue arrow. The scale bar represents 500 μm

HE staining is one of the most basic and commonly used staining methods in pathological experiments. The coloring of HE staining is closely related to the type of tissue or cell, physiological cycle, and pathological state [22]. Compared with the sham group, after 1, 3, 7, and 21 days of TBI injury, the nuclei of nerve cells in the impacted hemisphere were dark purple (marked by the blue arrow, Fig. 5b). The cell morphology shrank and the neurons with full morphology were significantly reduced (Fig. 5). The results showed that rubber bullet impact induced TBI caused pathological damage to the brain tissue on the injured hemisphere of the mice.

### Phosphorylation of Tau was elevated in the injured hemisphere

**Fig. 6:**
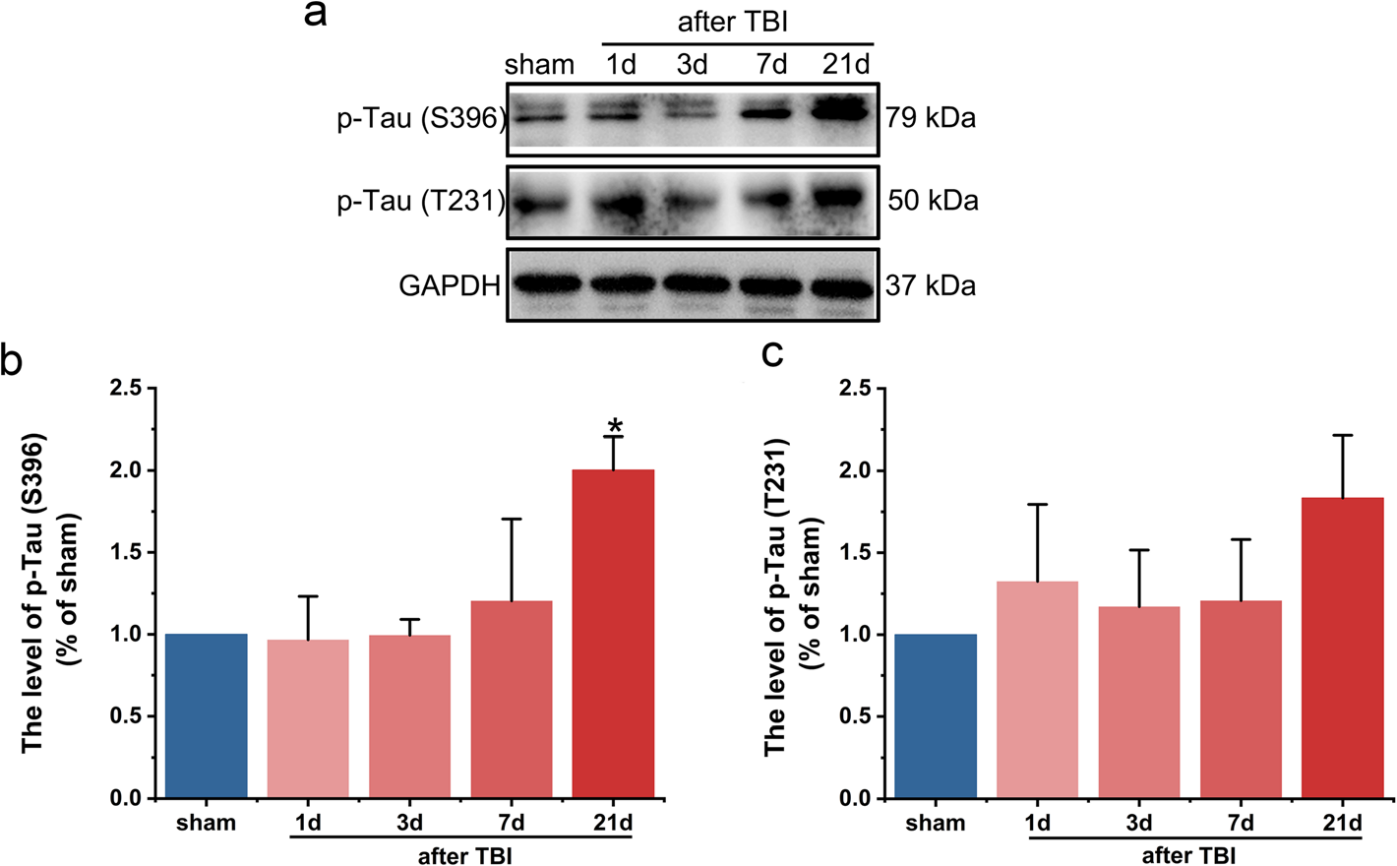
The effects of rubber bullet impact induced TBI on the levels of phosphorylated Tau protein. **a** Representative western blots of p-Tau (S396) and p-Tau (T231) in the cortex of the injured hemisphere. **b** The levels of p-Tau (S396) in the cortex of the injured hemisphere. **c** The levels of p-Tau (T231) in the cortex of the injured hemisphere. Data are presented as the mean ± S.E.M. and analyzed using one-way ANOVA; post hoc tests were conducted using Tukey’s test. *n* = 3/group. *P < 0.05 vs. sham group

The effect of rubber bullet impact induced TBI on the level of phosphorylated Tau was detected via western blot. TBI induced Tau phosphorylation at Ser396 epitope in a time-dependent manner, which was significantly increased 21 d after TBI compared with the sham group (*P* < 0.05, Fig. 6a, b). While the level of Tau phosphorylation at Thr231 epitope in the TBI group had an increasing trend but it was not statistically significant (Fig. 6a, c).

## Discussion

With the rapid development of modern society, road vehicles are increasing day by day, the construction industry is developing rapidly, and a large number of high-explosive weapons are applied in modern warfare. As a result, TBI is increasing, which has become an important global public health problem and a major difficulty for medical security. A good disease model is a basis for further research on the pathophysiological mechanism of TBI and its treatment measures. Although there are already some models, there is a need for animal models that are easier to operate and lower-cost. To meet this demand, the study describes a novel TBI model (rubber bullet impact induced-TBI), and the model was characterized.

Two weeks after injury, the TBI mice induced in this novel model showed cognitive dysfunction consistent with the symptoms observed in other TBI models [8–11]. Meanwhile, the effects were also consistent with observations of the effects of TBI in patients who have experienced repeated mild concussive injuries [23]. We have not yet systematically evaluated emotional function after subjecting mice to TBI [24], but this is a high-priority analysis that will be the focus of future experiments.

Another important finding was that the permeability of BBB was destroyed 48 h after the rubber bullet impacts induced-TBI. This finding is consistent with that of other TBI models, such as Shohami’s weight-drop model and controlled cortical impact injury [25]. The loss of integrity of the BBB is a serious event caused by TBI [26]. Since then, BBB is no longer a perfect protective barrier between the vascular septum and the brain, and its dysfunction leads to leakage of fluid, proteins, and immune system cells [27].

Many recent studies have shown that there were obvious injury and hemorrhage in brain tissue of TBI mice, and the neurons appeared stenosis, nuclear condensation, cytoplasmic hyperchromatism, and acute necrosis, which are typical TBI pathological features [28, 29]. In our study, the pathological features were reproduced in the novel TBI model.

Moreover, we interestedly found that the expression of phospho-Tau had been increased 3 weeks after impact. Tau protein is a member of the microtubule-associated protein family [30]. The phosphorylated tau aggregates further form neurofibrillary tangles (NFT), which have been considered a common pathological feature of many neurodegenerative diseases and TBI [31]. It’s reported that NFT was present in the mice brain after 3–4 months of chronic repetitive mild traumatic brain injury [32]. Whether NFT is present in our model still needs to be studied further with the addition of longer time points.

To keep stability of the TBI model, a few precautions should be taken into consideration. Firstly, age- and weight- matched mice are the best experimental materials and the low diversity in body weight is the principal precondition. Secondly, pre-experimentation in different age of mice to determine the number of shots is recommended. In our study, the 8 weeks of age mice survived 1 shot (mild), 2 shots (moderate), and 3 shots (severe) respectively. Finally, the critical steps and cautions in our protocol summarize the precautions during procedures, such as locating the impact point and the shooting angle. Regrettably, one limitation of present study is the TBI model we designed is still a preliminary conceptual model and lacks some parameter indications. Therefore, we still need to fine-tune the relevant parameters in the model, evaluate and optimize it in further study.

## Conclusion

Taken all together, histological, behavioral, neurological, and molecular data revealed that the rubber bullet impact induced TBI model mimics mild brain injuries in humans, such as BBB leakage, cognitive dysfunction, morphological damage, and tauopathy. This novel simple model of animal TBI is suitable for exploring pathobiological mechanisms as well as a screening of potential therapies for TBI.

## Supplementary Information

**Additional file 1.** The timeline of the experimental design.

## Data Availability

The datasets used and/or analyzed during the current study are available from the corresponding author on reasonable request.

## Abbreviations

TBI: Traumatic brain injury
BBB: Blood-brain barrier
EB: Evans Blue polymerase chain reaction
NFT: Neurofibrillary tangles
