# Supplementary figures and images for: A novel simple traumatic brain injury mouse model
Source: Chin Neurosurg J. 2022 Apr 1;8:8. doi: 10.1186/s41016-022-00273-5 (PMC8974042; doi:10.1186/s41016-022-00273-5)

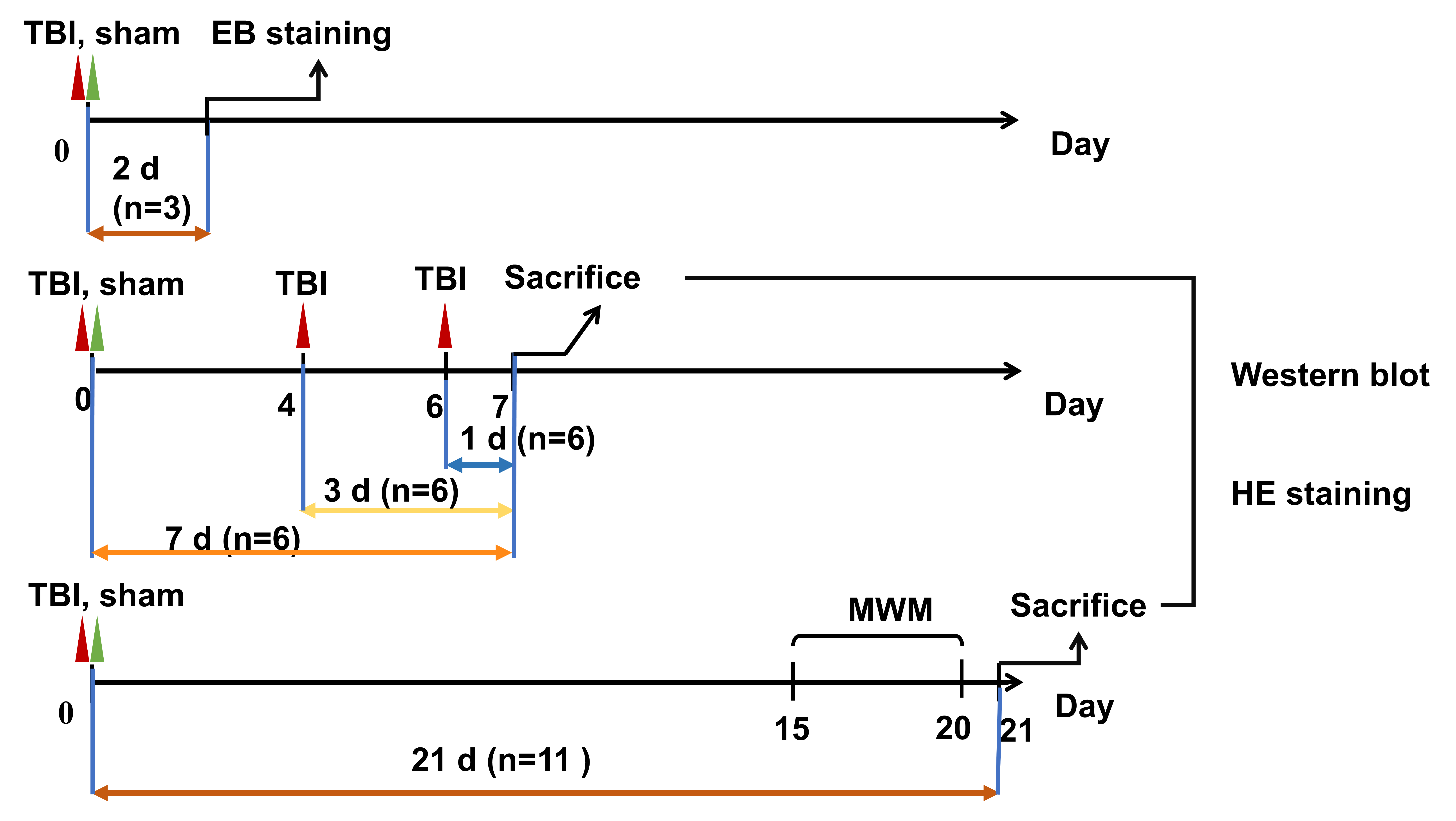

Supplement: Supplementary file 1 — Additional file 1. The timeline of the experimental design. [file 41016_2022_273_MOESM1_ESM.tif]
